# A thermodynamic assessment of the reported room-temperature chemical synthesis of C_2_

**DOI:** 10.1038/s41467-021-21433-8

**Published:** 2021-02-23

**Authors:** Henry S. Rzepa

**Affiliations:** Department of Chemistry, Molecular Sciences Research Hub, Imperial College London, White City Campus, 81 Wood Lane, London, W12 OBZ UK

**Keywords:** Chemical synthesis, Theoretical chemistry

**Arising from** K. Miyamoto et al. *Nature Communications* 10.1038/s41467-020-16025-x (2020)

Trapping experiments have recently been claimed^1^ to demonstrate the first chemical synthesis of the diatomic species C_2_ at room temperatures, using an alkynyl iodonium salt precursor. Here I explore the thermodynamic energies of the reaction using three different models, which concur in indicating that the reaction is endoenergic by > 40 kcal/mol. If C_2_ is indeed readily formed at such temperatures, a mechanism to counter this unfavourable thermodynamic energy must be identified.

The room-temperature chemical synthesis of C_2_ was first reported in the form of a pre-print^1^ and has now appeared as a full paper^2^. The core of the article asserts at its simplest that a transient intermediate **11** formed as ^**-**^C ≡ C–I^+^–Ph by treating precursor **1a** with a source of fluoride anion can fragment to singlet C_2_ and I–Ph at ambient or low temperatures (Fig. 1). The so-generated C_2_ can then be trapped in a variety of ways which are highly suggestive of this putative intermediate. Iodonium species are indeed known in the literature as alkynylation reagents^3^, albeit with a proposed mechanism of action for inserting C≡C into molecules that does not involve free C_2_. Most of the trapping experiments in the present article are reported in solution, with an implied assertion that singlet C_2_ as a discrete species is insufficiently reactive to be captured by solvent rather than by a chemical trap. One experiment is claimed to produce C_2_ gas, but in this case the implication is that the C_2_ is insufficiently reactive to be trapped by the fritted glass filter through which it must pass.

**Fig. 1:**
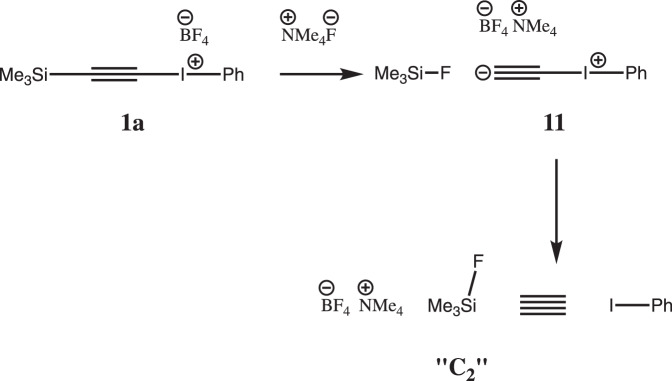
Reaction scheme for proposed^1^ chemical synthesis of singlet C_2_. The proposed^1^ chemical synthesis of singlet C_2_, in which an alkynyl phenyl iodonium salt is treated with fluoride anion to remove the trimethylsilyl group and generate an internal zwitterion **11**. This species is then proposed to fragment into iodobenzene and putative singlet C_2_.

One can subject the reaction sequence in Fig. 1 to a reasonableness check based on bond dissociation energies (BDEs). A more quantitative assessment is available through higher level quantum mechanics. The authors themselves have not commented^2^ on this aspect in their current article, which is based on purely experimental aspects. Addressing firstly the thermodynamics of the core equilibrium (Eq. 1)

1 $$^ - {\mathrm{C}} \equiv {\mathrm{C - I}}^{\mathrm{ + }} - {\mathrm{Ph}} \rightleftarrows {\mathrm{C}}\mathop { \equiv }\limits^{....} {\mathrm{C}} + {\mathrm{I}} - {\mathrm{Ph}}$$

Estimates of the experimentally derived BDE of the alkynyl-I bond in C≡C–I^+^–Ar iodonium salts are in the region of 70–80 kcal/mol^4–7^. When the iodonium C–I bond cleaves, it is directly replaced by a fourth bond as represented by C⩸C, the BDE of which is experimentally estimated at the much lower value of ~17 kcal/mol^8,9^. When allowance is made for a gain of ~10 kcal/mol of free energy resulting from increase in entropy, this implies that around 43–53 kcal/mol of bond energy must be recovered by the formation at ambient temperatures of C_2_ itself.

To assess this aspect more quantitatively, the ωB97XD/Def2-SVPD density functional method, with solvation energies estimated using a continuum method set for dichloromethane, has been applied to the reaction shown in Fig. 1 (with NMe_4_ replacing NBu_4_)^10^. This suggests that the relative free energies Δ*G*_298_ of **1a**, **11** and the assemblage labelled “C_2_” (C_2_ + I–Ph + Me_3_SiF + Me_4_N^+^BF_4_^−^) are 0.0, 0.1 and +68.2 kcal/mol, respectively. The computed energetics of C_2_ itself were calibrated against the two consecutive bond dissociation reactions (Eq. 2)

2 $${\mathrm{HC}} \equiv {\mathrm{CH}} \to {\mathrm{HC}} \equiv {\mathrm{C}}^{\mathrm{ \bullet }}{\mathrm{ + H}}^{\mathrm{ \bullet }} \to {\mathrm{C}}\mathop { \equiv }\limits^{....} {\mathrm{C}} + 2{\mathrm{H}}^{\mathrm{ \bullet }}$$

for which the thermochemistry has been determined in the gas phase^8,9^. This calibration suggests that the relative energy of C⩸C itself is too high by ~28 kcal/mol when computed using the ωB97XD DFT functional and the Def2-SVP basis set. If this correction is applied to the DFT results, then the computed free energy Δ*G*_298_ of the reaction **1a** → “**C**_**2**_” is reduced from +68.2 to ~+40 kcal/mol. This is in broad agreement with the simple argument advanced above from experimentally based BDEs (43–53 kcal/mol).

A further, simplified, model^11^ at the CCSD(T)/Def2-TZVPPD/SCRF = dichloromethane level was computed (Eq. 3).

3 $${\mathrm{Me - I}}^{\mathrm{ + }}{\mathrm{ - C}} \equiv {\mathrm{C}}^{\mathrm{ - }} \to {\mathrm{Me - I + C}}\mathop { \equiv }\limits^{....} {\mathrm{C}}{\mathrm{.}}$$

A solvation model is essential, since the ionic reactant is expected to be substantially stabilized by solvation compared to the non-ionic reaction products. At this level of theory, the energy of C_2_ itself is computed to be too stable by ~4.6 kcal/mol. With this correction applied, the overall reaction free energy emerges as Δ*G*_298_ + 47.1 kcal/mol, again in the range 43-53 kcal/mol. The former value corresponds to a half-life of a unimolecular reaction (Eyring theory) of ~10^18^ hours at 298 K.

To add further insights, CCSD(T)/Def2-TZVPP model studies^12,13^ were conducted in which the I^+^–Ph leaving group is replaced by what must be the ultimate leaving group He^+^, itself formed by radioactive decay of tritium. Here, unlike the C–I bond, the BDE of the C–He^+^ bond is tiny (~1 kcal/mol) and its replacement by C_2_ does indeed then result in a reasonably exo-energic equilibrium (ΔΔ*G*_298_ −42.2 kcal/mol), augmented again by entropy gain. This serves as a reminder that C_2_ itself is a very high energy species.

How can a reaction shown in Fig. 1 and generating the proposed free C_2_ overcome a reaction endo-energicity ΔΔ*G*_298_ of +47 kcal/mol, the most accurate estimate obtained by the computations reported here? Eyring theory tells us that at 298 K, unimolecular reactions with respectively a half-life of 1 min and 1 h correspond to free energy barriers of 20.0 or 22.5 kcal/mol, significantly lower than the range of energies predicted above. There are several possibilities to consider.

- Firstly, an as yet un-identified mechanism recovers the energy. Is it possible that sufficient enthalpy can be recovered by say reorganisation of ionic lattice energies so that the resulting free energy barrier Δ*G*_298_^‡^ could promote a sufficiently rapid (half-life <1 h) reaction at room temperatures? This would allow C_2_ to be trapped by another species, but this would compete with ~barrierless reverse trapping by Ph–I. If so, by what type of mechanism could this recovered energy then be concentrated directly into the carbon-iodine bond in order to cleave it?
- That the range of thermochemical values for the reaction obtained by the three models above is wrongly predicted to be too high by 20–25 kcal/mol.
- Alternatively, one might consider that free C_2_ itself is not produced, but instead some other species which must account for the results of the trapping reactions. A possible check on the gas-phase trapping would be to condense whatever species emerges from the dry reaction flask onto a cold-finger at liquid helium temperatures in an argon matrix and subject this directly to spectroscopic (Raman or other) analysis as an alternative to chemical trapping.

If the formation and trapping of C_2_ by the chemical reaction sequence shown above can indeed be independently confirmed, then this leaves us with a fascinating chemical challenge of how an otherwise apparently excessively endo-energic reaction can be promoted to viability. No solution to that perplexing dilemma is offered here.

## Data Availability

All relevant FAIR (Findable, Accessible, Interoperable, Reusable) datasets are available from a data repository^10,11^ via the collection DOIs 10.14469/hpc/5610 and 10.14469/hpc/7185 and datasets cited therein.
